# Who do we trust and how do we cope with COVID-19? A mixed-methods sequential exploratory approach to understanding supportive messages across 35 cultures

**DOI:** 10.1057/s41599-023-01747-2

**Published:** 2023-05-30

**Authors:** Algae K. Y. Au, Jacky C. K. Ng, Wesley C. H. Wu, Sylvia Xiaohua Chen

**Affiliations:** The Hong Kong Polytechnic University, Hung Hom, Kowloon Hong Kong

**Keywords:** Psychology, Cultural and media studies

## Abstract

Based upon a mixed-methods follow-up exploratory model, we examined the link between trust and coping during the early outbreak of the COVID-19 pandemic at the society level. Qualitative data were collected from the supportive messages written by 10,072 community adults across 35 societies. Trust and coping were used as the two pre-defined themes in the conceptual content analysis. Five subthemes emerged from the theme trust, depicting five distinct trusted targets: God, a larger us, country/government, science/healthcare, and the affected. Six subthemes emerged from the theme coping, depicting six distinct coping strategies: interpersonal/social coping, religious/spiritual coping, acceptance, blame, wishful thinking, and strength-based coping. A follow-up quantitative investigation also showed that four society-level factors (viz., individualism, cultural tightness, globalization, and severity of pandemic) had differential effects on people’s trusted targets and ways of coping with the pandemic. Our study made both methodological and practical contributions to cross-cultural research on COVID-19 by using a mixed-methods approach in a multinational study and demonstrating the importance of making meaningful virtual connection during a time of physical distancing.

## Introduction

Since the first quarter of 2020, the coronavirus (COVID-19) pandemic has sent shockwaves around the world (WHO, 2020). Besides economic and social disruption, the uncertainty and fear of such a novel infectious disease has also triggered psychological turmoil among global populations, creating intense stress and anxiety (Chen, Ng et al., 2021; Choi et al., 2020; Ismail et al., 2021; Sahashi et al., 2021). In a time of global panic, probably nothing is more important than having clear, strong, and trustworthy guidance and a belief that there is someone we can depend on, to give us confidence in doing the right thing, at the right time, and in the right way (Ahern and Loh, 2020; Thoresen et al., 2021). Meanwhile, if our trust is misplaced or abused in the midst of such circumstances of extreme uncertainty and vulnerability, the consequences can be negative or even deadly (Gisondi et al., 2022). Moreover, as the massive changes wrought by the pandemic have happened so rapidly and abruptly, it is equally important for us to have effective coping strategies to adapt and respond to the everchanging “new normal” of social isolation and new modes of working and living, and to deal with the accompanying negative emotions of frustration, sadness, anger, grief, and anxiety (Cheng et al., 2021; Coiro et al., 2021). Using improper coping strategies, however, can lead to negative psychological outcomes (Babicka-Wirkus et al., 2021). The present research focused on two important psychological concepts crucial for navigating the pandemic—trust and coping.

### Trust

Trust is a way of managing our dependencies on others and is “a window to the social reality” (Liu et al., 2018, p. 791) that helps us make sense of the world. It is a social practice that reflects our value of and feeling toward trusted targets, based on our rational and instrumental judgements and our relational and affective bonds with them (Flores and Solomon, 1998; Lewicki et al., 2006). Our trusted targets can be general or specific, and can be people, systems, or institutions that range from all humanity, family and friends, the community, and the government, to the almighty God, and so on, as long as we believe or feel that they have the ability, integrity, and benevolence that we may rely on (Mayer et al., 1995; Pirutinsky et al., 2020; Tabery and Pilnacek, 2021). During the COVID-19 pandemic, trust can be regarded as a social capital indicator, reflecting our sense of security and confidence in relying on others to buffer our feelings of powerlessness and helplessness in the face of such emergency and risk situations (Fersch et al., 2022; Li et al., 2022). Empirical findings have shown that trust is an important psychological construct that can shape the course of pandemic by influencing our interpretation of COVID-19 information (Filkuková et al., 2021; Gisondi et al., 2022), adherence to social distancing guidelines (Gratz et al., 2021), and timely treatment-seeking behavior (Antinyan et al., 2021). In other words, trust during the pandemic is a matter of life and death.

### Coping

Coping refers to the ways we deploy various cognitive and behavioral strategies to deal with stressful events to reduce its emotional toll on us (Lazarus and Folkman, 1984). Such psychological stressors can be actual harm or loss that have happened or are happening, or threat or challenge that are lying ahead (Lazarus, 2006). The level of perceived distress does not only depend on the objective magnitude of disruptiveness of the negative events, but also our ability to deploy proper stress-reduction strategies to make the subjective experience more manageable and comprehensible, or even more meaningful (Tedeschi and Calhoun, 1995). As the pandemic has created huge disruptions to nearly every aspect of our daily life and profoundly affected almost everyone across the globe, the concept of coping has also become a subject of interest in COVID-19 research, documenting how people around the world deployed different coping strategies to deal with such rapid and substantial changes, and how the use of these strategies were associated with the trajectories of those psychological distress (Fluharty et al., 2021; Shamblaw et al., 2021; Wang et al., 2022). In other words, how we cope with the COVID-19 stress could have profound consequences for our mental health, both short-term and long-term.

#### Trust and coping

The relationship between trust and coping has been examined in past literature. For example, individuals high in interpersonal trust are more likely to seek help from their social network and tend to cope better than those low in interpersonal trust (Grace and Schill, 1986; Schill et al., 1980). The mere expectancy of the trustworthiness of others can facilitate a positive interpretation of the reassurance and assistance provided, which in turn will enable individuals to benefit from and utilize the help more effectively and thus cope better (Grace and Schill, 1986). Potentially, trust may also shape our pandemic experience by influencing how we cope during this collective trauma. The relationships between trust and coping have been examined in some COVID-19 studies, showing that people around the world who had specific trusted targets to rely on were generally more able to cope with the pandemic. In the US, a higher trust in God was associated with lower stress among Orthodox Jews (Koning et al., 2021; Pirutinsky et al., 2020). In the Netherlands, teenagers who had mentors such as teachers and adult friends to rely on had fewer psychological difficulties (Koning et al., 2021). In Hubei, the epicenter of China’s pandemic, frontline healthcare staff relied on their trust in the medical system as a main source of strength to cope with the intensive work demands during the first outbreak (Ma et al., 2021). While evidence from existing COVID-19 literature has shown that trust and coping are related in many ways in different cultures, there is scarce published data that explicitly examine the pattern of trust and coping relationship across cultures in this global pandemic.

#### Trust, coping, and cultural, socioeconomic, and epidemiological factors

Given that trust is largely derived from social experiences whereas coping is essentially context-dependent, cultural characteristics of a society, which varies in its government’s responses to the pandemic, may impact our perception of trust and the ways we cope with challenges (Chen, Lam, et al., 2021). In the face of this unprecedented COVID-19 pandemic, people may use their cultural-specific values to guide their survival processes by deciding whom to trust and how to cope to ensure that they can come out of the pandemic safe and sound. The present study focused on two major cultural dimensions: individualism-collectivism (Hofstede, 2001) and cultural tightness-looseness (Gelfand et al., 2006; Gelfand et al., 2011). Individualism-collectivism refers to the preference for viewing oneself as an independent being and emphasizing personal interest vs. viewing oneself as a member of an ingroup and emphasizing group interest (Hofstede, 2001). Cultural tightness-looseness refers to the strictness of social norm adherence in terms of low personal liberty and high censuring pressure vs. high personal liberty and low censuring pressure (Gelfand et al., 2006; Gelfand et al., 2011). Some recent evidence suggesting that these two cultural dimensions may determine how people react to health measures (Chen, Frey, et al., 2021; Kemmelmeier and Jami, 2021; Luo et al., 2022; van Hoorn, 2015) and deal with stress (Burkova et al., 2021) during the COVID-19 pandemic. For example, cross-cultural data from 111 countries revealed that people in individualistic cultures were less abiding by the official lockdown rules (Chen, Frey, et al., 2021), whereas data from 45 US states found that respondents from tighter states, as compared to looser states, were more likely to trust government officials and regard the preventive measures of wearing mask as a civic duty rather than an infringement of personal freedom (Kemmelmeier and Jami, 2021). Moreover, data from a 23-culture comparison during the first wave of pandemic in 2020 found that individualism was positively, whereas cultural tightness was negatively, associated with anxiety (Burkova et al., 2021). Given the unprecedentedness of Covid-19, more empirical evidence is needed to further ascertain the roles that the two cultural dimensions may play in pandemic trust and coping.

Besides, socioeconomic factors such as globalization, referring to the degree of social and economic exchange across geographical and political boundaries (Keohane and Nye, 2020), may also impact people’s behavioral responses to COVID-19 (Chen et al., 2023), trust (Polillo, 2012) and coping (Sharma and Sharma, 2010). Increased international economic competitions arising from globalization may diminish social trust toward unknown others (Polillo, 2012). When a country becomes more globalized, the function of traditional values as anxiety buffer may also be weakened, leaving people with fewer options and resources for coping (Sharma and Sharma, 2010). Using data from 149 countries, a recent study found a positive association between globalization and accumulated COVID-19 fatality rate, providing initial empirical support for the potential role of globalization in the COVID-19 pandemic outcomes (Farzanegan et al., 2021). So far, however, discussion on the effects of globalization on trust and coping in the COVID-19 pandemic context remains limited.

Moreover, being a novel, extraordinary, and emotionally charged social experience, the impact of the COVID-19 pandemic itself on trust and coping cannot be overlooked. Some interesting cultural differences in the change of trust during the pandemic were observed. For example, a global survey conducted in the summer of 2020 revealed that nearly 30% of US participants reported that their religious faith had been bolstered by the pandemic, a phenomenon that was not seen in other advanced economies (Pew Research Center, 2021). Another large-scale longitudinal survey also found large variations in the levels of trust held for scientists, government, and others, across 12 countries and between the timepoints of March and December 2020 (Algan et al., 2021). The relationships between pandemic severity and coping were intriguing, but inconclusive. For example, one study showed that during the early outbreak in Italy, children in high-risk areas used fewer task-oriented but more emotion- and avoidant-oriented coping strategies than children in medium- or low-risk areas (Liang et al., 2020). However, a comparative study among three European countries (Italy, Germany, and Austria) during the early phase of the first lockdown in each country found that respondents from Italy, the country that was most severely hit, reported lower use of positive and negative coping strategies than respondents from the other two countries in which the COVID situation was less severe (Eichenberg et al., 2021). Taken together, cultural, socioeconomic, and epidemiological factors might have played crucial roles in trust and coping strategies during the pandemic that need to be further examined.

There is a growing literature on trust and coping during the period of COVID-19, and it mostly adopts a quantitative approach that is limited in capturing people’s contextualized experiences in perceiving and dealing with the challenges brought by the pandemic (Teti et al., 2020; Tremblay et al., 2021). To complement this, we adopted a mixed-methods sequential exploratory approach in this study. A sequential exploratory design is a two-phase model that starts with a qualitative phase of collecting and analyzing qualitative data (in this research, supportive messages posted by community adults) to identify conceptual themes, which are then sequentially used in the quantitative phase for further exploration (Creswell and Clark, 2017). This research goes further and then integrates the qualitative and quantitative data to offer more insights and enrich our understanding of the unique, complex, and dynamic pandemic experiences, in both depth and breadth.

## Qualitative phase

Based upon the literature review, our qualitative inquiry aimed to answer the following two research questions:

RQ1: Who were the trusted targets and how they were trusted in the face of the COVID-19 pandemic?

RQ2: What were the coping strategies and how they were used to cope with the COVID-19-related stress?

To determine the targets of trust and coping strategies, we collected and analyzed open-ended supportive messages posted on an online panel. Supportive message writing is a communicative behavior with the primary goal of acknowledging the feelings of and providing reassurance and encouragement to the distressed others (High et al., 2019; Kim et al., 2011). Nevertheless, writing a supportive message is a complex and cognitively demanding process (MacGeorge et al., 2011). To craft a supportive message that meets the specific needs of the distressed others, the writers have to infer the target recipients’ thoughts and feelings to understand the proximal causes of the distress (Burleson, 1985; Burleson et al., 1994; High et al., 2019; MacGeorge et al., 2011). Such inference is essentially egocentrically biased, involving the use of one’s own experience as a frame of reference, and the projection of one’s own values and affects onto the experience of others (Mitchell, 2009; Samson et al., 2010; Trilla et al., 2021). Following this line of reasoning, we regard the supportive messages in the current research as proxies of the message writers’ own cognitive and affective response to the fear and uncertainty that the COVID-19 pandemic had brought.

## Methods

### Participants and procedure

Supportive messages were collected from 10,072 community adults for the period of 9–20 April 2020, from 35 societies across Asia, Europe, North America, South America, Oceania, and Africa, as part of a large-scale research project involving 18,171 participants on an online panel (Chen, Ng et al., 2021). Informed consent was obtained from all participants at the beginning of the study, indicating their willingness to participate in the study. Participants were warranted the anonymity of their participation and their right to withdraw; and were assured that all data collected would be securely stored in encrypted files, a practice that was in line with the data storage policy of the authors’ institution. Participants also reported demographic information at the end of the study. Ethics approval was obtained from the Human Subjects Ethics Sub-committee of the authors’ institution. We included the open-ended question *“Would you like to write a few words to communicate support and encouragement for individuals who are affected by the coronavirus?”* in the online survey, asking participants to indicate their willingness/unwillingness to write a message by choosing the appropriate “yes or no” option. Those who chose “no” went directly to the next question in the questionnaire. Those who chose “yes” were directed to an expandable text box to write a supportive message. We used an expandable text box to allow participants to write as much as they wanted, without the constraint of length restrictions (Decorte et al., 2019). After writing the message, participants were then directed to the next question to continue with the questionnaire. To ensure that participants were paying full attention throughout the study (Maniaci and Rogge, 2014), three directed questions were included in the survey for attention check (e.g., “This is a control question. Select “Agree” and move on”). Participants’ responses were considered as valid only if they passed all three directed questions. A final sample of 10,072 (55.43% of the total sample) took the time to write supportive messages. The final sample was comprised of 50.1% females, 49.9% males, with a mean age of 42.49 years and an age range of 18–89. The majority of the sample (70.3%) were employed, while 15.2% were unemployed or retired, 8.1% were students, and 6.3% were homemakers. Most participants (60.9%) were university educated and had a bachelor’s degree or above, 35.3% had completed high school, while 1.8% had not finished high school. The response rate was highest in the Philippines (87.84%) and lowest in Finland (27.41%).^1^ A breakdown of the final sample is presented in Table 1.

**Table 1:** Sample size per society and the number and percentage of respondents writing supportive messages.

	Sample size	Respondents who wrote encouraging messages	
		*N*	%
Argentina	522	294	56.32
Australia	515	208	40.39
Brazil	530	317	59.81
Canada	526	180	34.22
China	519	299	57.61
Egypt	516	302	58.53
Finland	518	142	27.41
France	523	203	38.81
Germany	528	148	28.03
Hong Kong	526	271	51.52
India	519	376	72.45
Indonesia	526	439	83.46
Italy	527	287	54.46
Japan	515	218	42.33
Malaysia	525	335	63.81
Mexico	525	348	66.29
Netherlands	511	175	34.25
New Zealand	507	196	38.66
Nigeria	516	408	79.07
Pakistan	518	374	72.20
Philippines	510	448	87.84
Portugal	511	216	42.27
Russia	517	230	44.49
South Africa	523	306	58.51
South Korea	523	381	72.85
Singapore	524	308	58.78
Spain	518	332	64.09
Sweden	510	155	30.39
Taiwan	515	285	55.34
Thailand	516	386	74.81
Turkey	515	348	67.57
UAE	525	370	70.48
UK	523	206	39.39
USA	516	186	36.05
Vietnam	513	395	77.00
Total	18,171	10,072	55.43

### Data analysis

#### Translation

Thirty-five datasets in 23 languages were created from the 35 societies. To ensure an accurate interpretation of the meaning of data that truly reflect the perspectives of participants, all non-English messages were translated into English by professional translators who were familiar with both the native languages and the local culture of specific societies. The translated messages were further checked by the first and third authors, and any grammatical or linguistic issues were clarified and resolved with the translators before merging the messages into a single dataset. After translation, the 10,072 messages yielded a total of 128,757 words. The average message length was 12.78 words, ranging from brief messages with one or two words to more elaborative messages up to 181 words long.

#### General analytical approach

We integrated reflexive thematic analysis (Braun and Clarke, 2006) and conceptual content analysis (Berelson, 1952) in our analytical approach. Reflexive thematic analysis was used to help us identify the patterns of meaning across the messages, whereas conceptual content analysis allowed us to quantify the occurrence of specific themes and subthemes in the messages for statistical analysis in our quantitative phase.

Microsoft Excel version 2210 was used in the qualitative analysis. Analyst triangulation was used to ensure reliability of data (Patton, 1999). Specifically, three coders (first author, third author, and a research assistant with a master’s degree in psychology) independently analyzed the same qualitative data in the reflexive thematic analysis, starting with Argentina, which was the first on the list of the 35 societies in ascending alphabetical order. The coding was directed toward our primary research questions, focusing on the themes of *trust* and *coping*. We followed Braun and Clarke’s (2006) analytical process of getting familiar with the data, generating codes, and defining and revising the subthemes. We coded each respondent’s message as the unit of analysis, allowing multiple codes for each message. Subthemes identified from the coding were then organized into a coding frame, and a recursive process of coding and codebook modification was conducted with all remaining societies. After the codebook revision was completed, the entire dataset was coded again using the finalized codebook. To ensure trustworthiness of coding, we reflected and discussed on how and why specific messages were coded, throughout the coding process. To minimize selective perception and interpretive bias, we further examined the data reliability with Fleiss’ Kappa (Fleiss, 1971) to statistically compare the findings across three coders. Due to our large sample size, a subsample of 1,164 messages (11.56%) were randomly selected to test for interrater reliability (O’Connor and Joffe, 2020). The average of Fleiss’ Kappa was 0.86, ranging from 0.67 to 0.99 across subthemes, indicating a high level of rater agreement (Cohen, 1960; Landis and Koch, 1977).

#### The coding frame

Our final coding frame consisted of two pre-defined themes: *trust* (Theme 1) and *coping* (Theme 2), and eleven subthemes that emerged from the coding. Five subthemes were derived from Theme 1, and each depicted a specific trusted target: (i) God, (ii) us, (iii) country/government, (iv) science/healthcare, and (v) those affected. Six themes were derived from Theme 2, and each depicted a specific coping strategy: (i) interpersonal/social coping, (ii) religious/spiritual coping, (iii) acceptance, (iv) blame, (v) wishful thinking, and (vi) strength-based coping (see Table 2). Details of the themes and subthemes are presented in the findings section.

**Table 2:** Numbers and sample messages of subthemes.

Themes and subthemes	Numbers (per thousand)	Sample messages
*Trust*		
Trust in God	89.46	Contracting the virus is an act of God and definitely God will heal you.
Trust in a larger us	6.55	Keep it up, human can defeat the virus.
Trust in country/government	7.64	No need to worry… Our UAE will do its best to protect us.
Trust in science/healthcare	27.60	Have faith in medical advances, there will soon be a cure for this coronavirus epidemic.
Trust in those affected	7.45	Believe that you can overcome this.
*Coping*		
Religious/spiritual coping	53.02	I am praying for you. May God bless you all.
Interpersonal/social coping	181.39	Let’s work hard to fight against the epidemic.
Strength-based coping	629.77	Stay strong and maintain a positive mindset.
Wishful thinking	349.29	This all shall pass.
Acceptance	11.91	No one ever chooses to get ill; it happens.
Blame	1.99	I believe this is a man-made situation and I think that now US has started this biological war.

## Results

### Theme 1: Trust

Messages under this theme focused on putting faith in someone or something to overcome the pandemic crisis, highlighting one or more of the characteristics of the trusted targets in terms of ability, benevolence, and integrity, or, in some cases, legitimacy. Five subthemes emerged from this theme:

#### Subtheme 1.1: Trust in God

Messages under this subtheme focused on encouraging others to trust in God for divine protection, emphasizing God’s ability to protect and his benevolence. Respondents believed that the divine power was *“greater than this coronavirus”* (Female, aged 24, Hong Kong), that he could *“eliminate”* the pandemic (Female, aged 59, Philippines), *“cure”* (Male, aged 41, Mexico) and *“save”* (Female, aged 24, Hong Kong) the infected, and ultimately make them *“free again”* (Female, aged 59, Philippines). Respondents also believed that the supreme being was merciful, and *“loved”* (Female, aged 58, Pakistan) and *“looked after”* (Male, aged 41, Mexico) them, and would *“see* [them] *through”* (Female, aged 34, Nigeria) the pandemic. In this subtheme, the term “God” was broadly used to refer to a divine being, the sacred reality, or ultimate truth as perceived by the respondents. Previous literature revealed that trust in God could buffer uncertainty and facilitating a sense of meaning and purpose (Rosmarin et al., 2011). Research on COVID-19 also found that trust in God is positively associated with less stress and more perceived positive impacts of the pandemic (Pirutinsky et al., 2020).

#### Subtheme 1.2: Trust in a larger us

Messages under this subtheme mostly stressed trust in the collective effort in fighting the pandemic. Expressions such as *“global”* (Male, aged 42, Brazil), *“humans”* (Male, aged 32, China), *“everyone”* (Female, aged 39, Japan), or *“we”* (Male, aged 32, UAE) were used to describe this kind of collective identity. The severity of the COVID-19 pandemic in such a global scale implies a sense of common destiny that cannot be curbed with one’s individual efforts but the cooperation of others (Pagliaro et al., 2021). Placing their trust in a larger us can thus help people reduce their sense of vulnerability as individuals and increase their sense of control as a group during the pandemic, by believing that they can rely on each other in such collective fight (Thoresen et al., 2021).

#### Subtheme 1.3: Trust in country/government

Messages under this subtheme mainly focused on trust in country or government in terms of ability and integrity. Respondents believed that the country or government had the power to *“definitely overcome the epidemic”* (Female, aged 36, China), *“beat the pandemic”* (Male, aged 28, Vietnam), and *“win in this fight”* (Male, aged 36, UAE). The country or government was dedicated to *“looking for a cure”* (Male, aged 38, Mexico), “*doing* [their] *best to protect”* the people (Male, aged 25, UAE), and *“doing everything they can to ease the burden for all”* (Female, aged 41, Australia). However, some messages simply stressed that one should *“believe in the country, believe in the government”* (Female, aged 64, China) without justifying why, whereas some emphasized the legitimacy of the government as they were the *“authority”* (Female, aged 22, Canada) and in the position to make decisions for all, adding that it was the obligation of the people to *“be obedient”* (Female, aged 38, Philippines), *“comply with the regulations”* (Male, aged 37, Indonesia), and *“follow the instructions”* because they should not *“bite the hand that feeds [them]”* (Male, aged 57, Turkey). During the COVID-19 pandemic, whether trust in country/government is adaptive or not remains contestable. While trust in government was positively linked to compliance with social distancing measures (Gratz et al., 2021), the same type of trust could also, paradoxically, undermine the public’s risk perception and lower their compliance with the government’s risk management measures (Wong and Jensen, 2020).

#### Subtheme 1.4: Trust in science/healthcare

Messages under this subtheme stressed trust in science and healthcare professionals and systems in terms of ability, benevolence, and integrity. Respondents believed that science would *“prevail against this virus”* (Male, aged 62, Turkey) and that *“a cure”* and *“a vaccine”* (Female, aged 44, Brazil) would be found soon to *“put an end to the pandemic”* (Female, aged 31, Argentina), and that the patients were in the “*best possible hands*” (Female, aged 20, Spain) as the healthcare professionals would *“solve”* the pandemic *“problem”* (Female, aged 62, France). Respondents believed that healthcare professionals would provide *“care”*, *“comfort”*, and *“sympathy”* to the patients (Female, aged 20, Spain), perhaps even *“putting their own lives at risk”* (Male, aged 35, Turkey). Scientists and healthcare professionals were also believed to be fully dedicated, *“working day and night”* to *“save”* the people (Male, aged 40, Egypt) and *“doing everything they can”* to *“fix”* the pandemic (Male, aged 27, Portugal). Healthcare professionals and systems are usually regarded as the most trusted targets in previous pandemics and outbreaks due to their professional role in disease treatment (Li et al., 2016; So et al., 2004). During the COVID-19 pandemic, trust in healthcare and science has usually found to be beneficial in shaping people’s adaptive behavioral responses in terms of compliance with evidence-based prevention guidelines (Chan et al., 2020; Plohl and Musil, 2021) and treatment-seeking behavior (Antinyan et al., 2021). There were exceptions, though, such as trust in the Centers for Disease Control and Prevention (CDC), i.e., the public health agency of the US, was found to be negatively associated with adherence to social distancing guidelines (Gratz et al., 2021).

#### Subtheme 1.5: Trust in those affected

Messages under this subtheme focused on the belief that the affected had the ability to defeat COVID-19 themselves, though some messages did not specify what that ability was, but merely mentioned that the infected was *“capable of fighting the virus in their own way”* (Female, aged 48, France). In contrast, some messages explicitly emphasized the natural healing power of the body (Male, aged 30, UAE) and coined such power as *“the doctor within”* (Male, aged 64, Malaysia). At the time of data collection (April 2020), there was so much unknown about coronavirus and so much uncertainty around when and how one would contract that novel, deadly, and infectious disease, or to what extent and for how long one’s life would be changed by the pandemic. We reasoned that trust in those affected might be regarded as a means for the respondents to ward off their own fear of the coronavirus by convincing themselves that the disease can be defeated. Such self-efficacy has found to be important in buffering perceived COVID-19 related stress (Meyer et al., 2022).

### Addressing research question 1

The qualitative data of Theme 1 and its subthemes revealed that participants across 35 societies relied on five trusted targets (God, a larger us, country/government, science/healthcare, and those affected) to deal with the unknown and uncertainty during the outbreak. Their diverse choice of trusted targets reflected their unique way of managing their dependencies on specific others to alleviate their feelings of powerlessness and helplessness in such the pandemic situations.

### Theme 2: coping

Messages under this theme focused on deploying various strategies to deal with the stress of the pandemic. Six subthemes emerged from this theme:

#### Subtheme 2.1: Interpersonal/social coping

Messages under this subtheme focused on connectedness within a supportive network as a source of strength to cope with the pandemic challenges. Some messages emphasized family and friends, urging others to *“think of the lovely times”* they spent with their loved ones and assuring them that they would *“hug each other again”* (Female, aged 45, Italy). Some focused on communal support, considering the infected as *“part of [the] community”* and the whole community was *“eager for [them to] come back”* (Male, aged 49, Malaysia). Others stressed unity within the country, emphasizing that *“the country is with [the affected]”* (Male, aged 62, India) and that everyone should *“be united as a country”* (Female, aged 44, Singapore). Many focused on a broader sense of connectedness with the whole world, saying that *“everyone in the world” was “cheering”* for them (Female, aged 25, Taiwan), *“waiting for [their] fast recovery”* (Female, aged 28, Philippines), seeing them as *“family”* (Male, aged 33, Canada), and being *“like a real team”* (Male, aged 49, Argentina). A recent qualitative study also evidenced that having extensive supportive networks is helpful for coping with the emotional and practical challenges during lockdown (van Bortel et al., 2022).

#### Subtheme 2.2: Religious/spiritual coping

Messages under this subtheme focused on coping with the pandemic-related stress by finding relief, comfort, and meaning in the belief of the existence of a supernatural being (Pargament et al., 2005), through reframing the adversity as a trial from a higher power and an opportunity for spiritual growth that *“GOD [would] not test his servants if they [were] not strong”* (Male, aged 27, Malaysia), encouraging the use of religious rituals to find peace in the distressing situation that they should *“Keep praying to God and keep up the spirits”* (Female, aged 30, Indonesia), and calling for self-transcendence to reflect upon the global crisis as a spiritual reminder to *“spark compassion for others in the world”* (Female, aged 32, Brazil). Although the term “religion” often depicts the institutional aspect of worshiping a supernatural being, while “spirituality” generally describes the individual experience of personal transcendence, what is in common between the two terms is they both involve a search for a divine being, the sacred reality, or ultimate truth (Hill et al., 2000; Pargament, 2001). In this subtheme, we used the collective term “religious/spiritual coping” to refer to the use of benevolent religious reappraisals and spiritual support to find comfort, meaning, and strength in the adversity (Pargament, 2001; Park, 2007). Recent research found that religious/spiritual coping is especially important during the COVID-19 pandemic for people who were struggling through lockdown or coping with grief or loss (van Bortel et al., 2022).

#### Subtheme 2.3: Acceptance

Taking a somewhat pessimistic view, messages under this subtheme focused on accepting that the pandemic was *“out of control”* (Female, aged 31, Argentina) and such unpredictability was *“part of life”* (Male, aged 69, Singapore). The use of acceptance coping means that participants have resigned from striving for control, as they believe that the aversive situation is uncontrollable, the future is hopeless, and further action are fruitless (David and Suls, 1999; Nakamura and Orth, 2005). Such passivity can also spill over to other aspects of life during the pandemic. For example, a recent study found that during the early lockdown, individuals who passively accepted the COVID situation as unchangeable spent their time scrolling through social media platforms and sleeping, rather than using the time to improve themselves by learning new skills online or picking up old hobbies (Rishi et al., 2021).

#### Subtheme 2.4: Blame

Messages under this subtheme focused on blaming people, or an authority or government for causing, spreading, or poor handling of the pandemic. Some of the messages criticized people as *“selfish”* who *“should be jailed”* (Male, aged 71, UK) and were *“idiots”* and *“morons”* and *“a complete waste of space”* who should not *“deserve any sympathy”* (Female, aged 61, Singapore). Other messages focused on blaming the government or authority for failing to tackle the pandemic crisis, saying the *“crazy situation”* was *“mismanaged by many people in authority”* (Male, aged 38, US) and that people had to *“keep fighting”* because the government was *“stupid”* (Female, aged 57, Thailand). In fact, the COVID-19 pandemic has been coined the “pandemic of blame” (Bouguettaya et al., 2022, p 1) as people try to allocate responsibility for the cause and spread of the novel, deadly, and infectious disease to specific countries, governments, or groups (Bouguettaya et al., 2022; Yan et al., 2022). Attributing blame to others is generally regarded as maladaptive, as it implies a sense of uncontrollability that one’s own misfortune is at the hands of others (Thompson, 1985). However, identifying the “wrongdoers” and shifting the responsibility toward them may somehow protect one’s own ego (Freud, 2018), especially during the pandemic when people are struggling to make sense of the world that has been turned upside down (Bouguettaya et al., 2022). Indeed, a recent mixed-methods study found that in the US, more than half of the East Asian respondents had experienced various forms of racial microaggressions during the pandemic as they were subject to groundless accusation of being responsible for spreading the disease (Yan et al., 2022).

#### Subtheme 2.5: Wishful thinking

Messages under this subtheme focused on a reliance on hope rather than facts, evidence, and rationality to cope with the pandemic stress. Some messages attempted to induce false hope in the situation by viewing the adversity as transitory, using phrases such as “*this too shall pass*” and there would be a *“better world”* (Female, aged 62, Argentina) or *“nothing lasts forever”* so one should look forward to a *“better tomorrow”* (Male, aged 63, Singapore). These messages did not explain how or why the situation would improve, despite, at the time of data collection (April 2020), knowledge on this novel infectious disease being extremely limited—the spread of COVID-19 was out of control, the vaccine was still under clinical trial, and there was no cure. Using wishful thinking as a coping strategy means that participants fantasize a better future while avoiding or suppressing thoughts about the pandemic reality that is simply too overwhelming to deal with. However, despite the initial intent of reducing stress, wishful thinking has found to increase people’s level of anxiety during the COVID-19 pandemic instead (Wu et al., 2022).

#### Subtheme 2.6: Strength-based coping

Messages under this subtheme focused on encouraging the use of one’s inherent strengths and capacities to cope with the pandemic. However, most of the messages were slogan-like, lacking elaboration, such as: *“Hang in there!”* (Male, aged 48, Netherlands), *“Fight with all you’ve got!”* (Female, aged 34, Nigeria), or *“Don’t give in to this virus!”* (Female, aged 59, Egypt). Yet some messages managed to offer some practical advice to strengthen one’s ability to cope with the pandemic stress such as doing *“exercise”* (Female, aged 40, Hong Kong), or practice *“mindfulness”* and *“meditation”* (Female, aged 38, Australia). Strength-based coping reflects participants’ positive responses toward the COVID stress as they believe they can draw on their personal strengths, skills, and resources to cope with adversity (Waters, 2015). For example, strengthening one’s physical health through exercises such as walking, cycling, and home workouts has found to be one of the most common coping strategies people used during the pandemic (van Bortel et al., 2022).

### Addressing research question 2

The qualitative data of Theme 2 and its Subthemes revealed that participants across 35 societies deployed six coping strategies: interpersonal/social coping, religious/spiritual coping, acceptance, blame, wishful thinking, and strength-based coping to cope with the pandemic stress during the early outbreak. These coping strategies, though not necessarily adaptive, reflected participants’ desperate efforts in trying to reduce the emotional toll of the pandemic on them.

## Quantitative phase

Based on the five subthemes of trust and six subthemes of coping derived from the qualitative phase, we used SPSS 28 to perform quantitative analysis at the society level, with four objectives.

1. Examining the associations among five trusted targets during the pandemic, for example, whether societies that entrusted their fate to country/government are more likely to entrust their fate to science/healthcare.

2. Examining the associations among six ways of coping during the pandemic, for example, whether societies that utilized more interpersonal/social coping are more likely to employ wishful thinking.

3. Examining the associations between trusted targets and ways of coping, for example, whether societies that entrusted their fate to God are likely to use religious/spiritual coping).

4. Identifying society-level associates, that is, epidemiological, socioeconomic, and cultural factors, for the trusted targets and coping strategies.

### Methods

#### Subjects

**Fig. 1:**
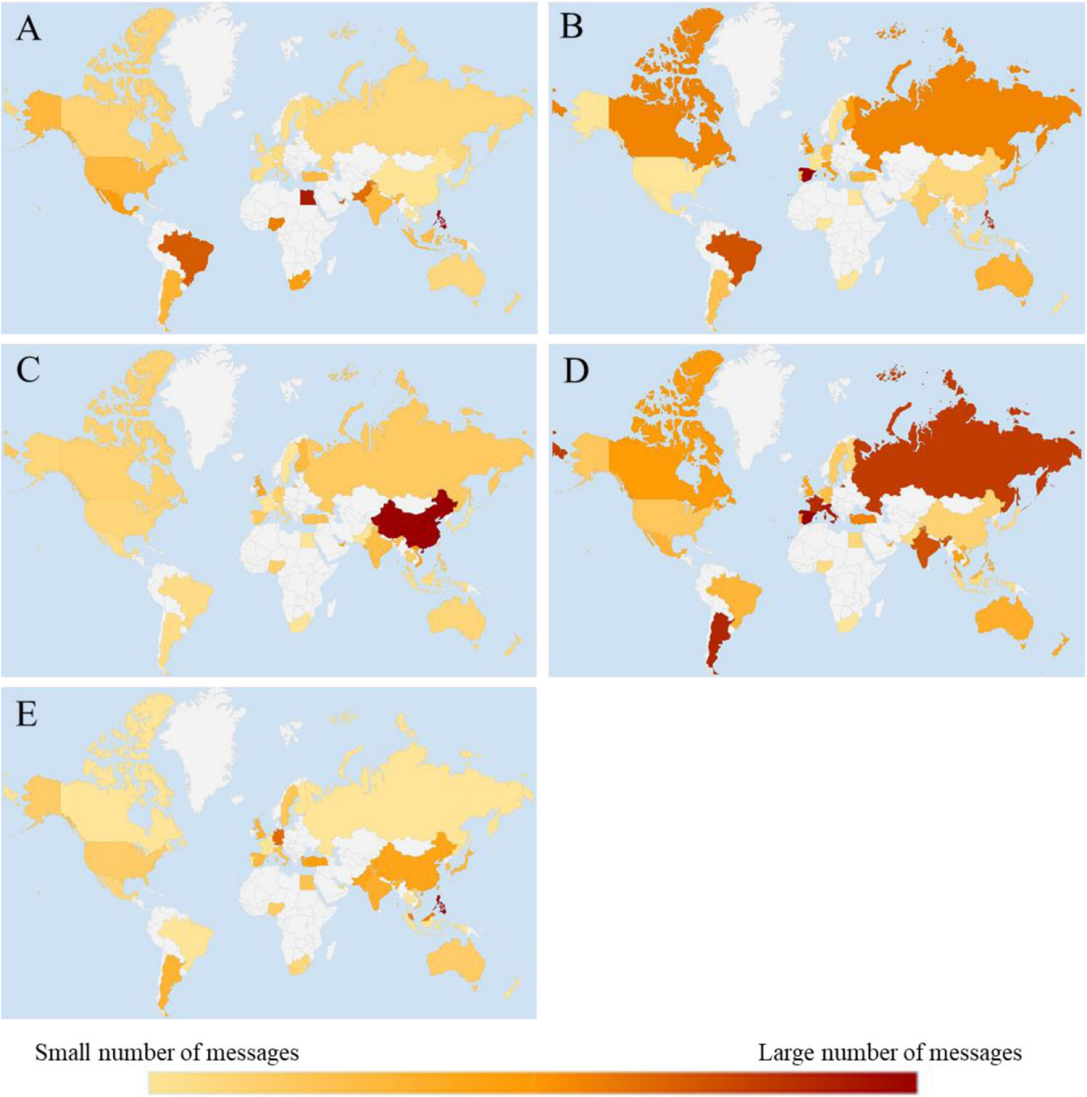
The choropleth maps that summarize the number of messages per thousand in five subthemes of trust across 35 societies. **A** Trust in God, **B** Trust in a larger us, **C** Trust in country/government, **D** Trust in science/healthcare, and **E** Trust in those affected.

**Fig. 2:**
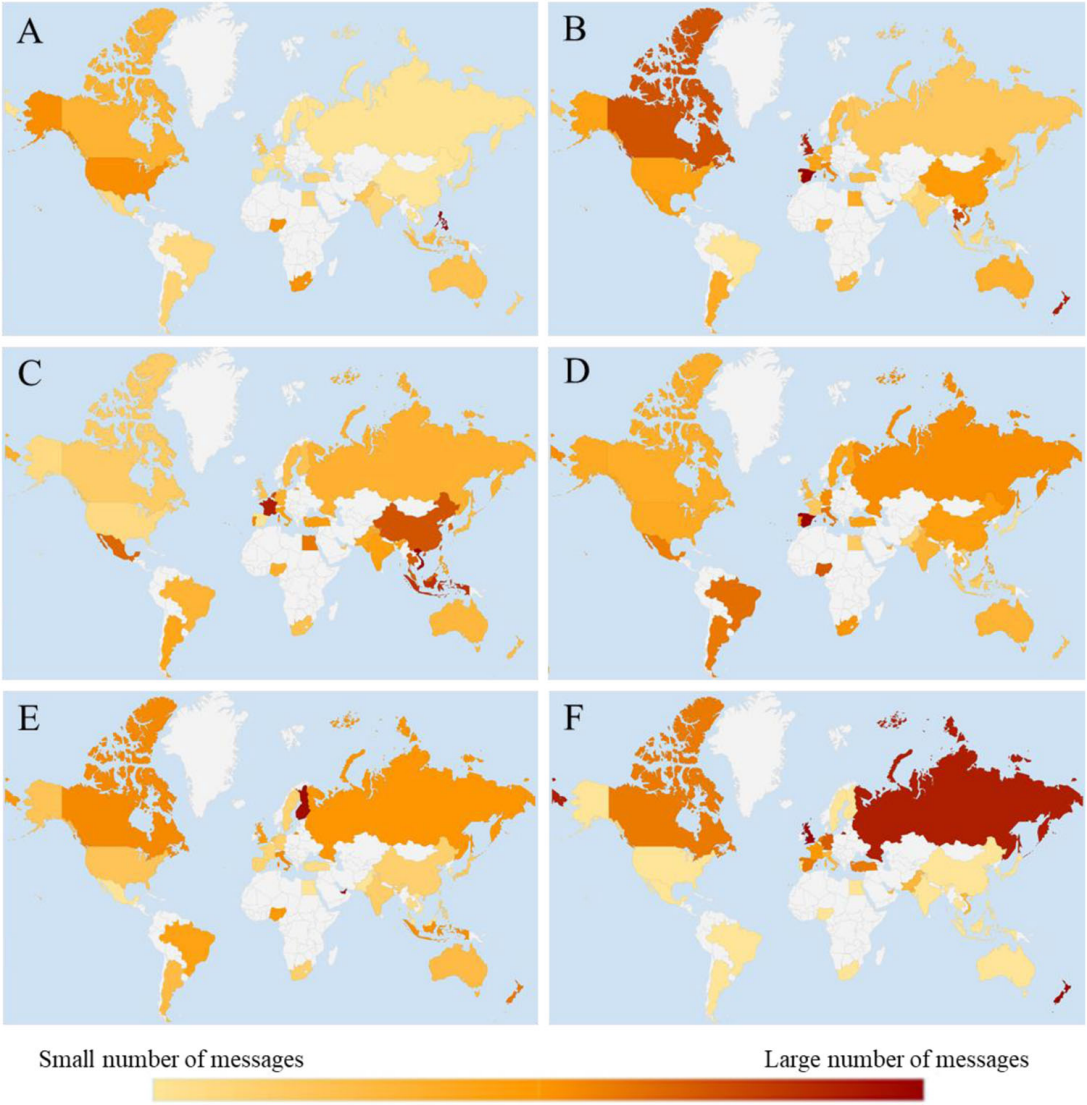
The choropleth maps that summarize the number of messages per thousand in six subthemes of coping across 35 societies. **A** Religious/spiritual coping, **B** Interpersonal/social coping, **C** Strength-based coping, **D** Wishful thinking, **E** Acceptance, and **F** Blame.

Based on the 10,072 supportive messages collected from 35 societies, we counted the number of themed messages for each society according to the 11 subthemes derived from the qualitative phase. The numbers of messages per thousand in each of the subthemes across 35 societies have been summarized in Figs. 1 and 2.

#### Measures of society-level variables

To identify the society-level associates for the five trusted targets and six coping strategies, we studied four society-level indices, namely the total number of confirmed cases (per 100,000 population) as an epidemiological factor, the KOF Globalization Index as a socioeconomic factor, and individualism (vs. collectivism) and cultural tightness (vs. looseness) as two cultural factors. Data on COVID-19 severity of infection were obtained from the European Centre for Disease Prevention and Control (ECDC). Data on the latest version of the KOF Globalization Index were obtained from Gygli et al. (2019). Data on the cultural dimensions of individualism (vs. collectivism) and cultural tightness (vs. looseness) were obtained from Hofstede (2001) and Gelfand et al. (2011), respectively.

### Results

#### Associations among trusted targets and coping strategies

To achieve the first and second objectives, we computed the correlations among the numbers of themed messages across five trusted targets (Table 3) and the correlations among the numbers of themed messages across six ways of coping (Table 4). First, there was a positive correlation between trust in science/healthcare and trust in a larger us, *r* = 0.49, *p* = 0.003, indicating that societies that trusted in science/healthcare were more likely to trust in the ability of collective efforts to fight the pandemic. Second, the use of strength-based coping was negatively correlated with the uses of both wishful thinking, *r* = −0.48, *p* = 0.003, and acceptance, *r* = −0.45, *p* = 0.007. Interestingly, we found a positive correlation between the uses of interpersonal/social coping and blaming, *r* = 0.53, *p* = 0.001, revealing that societies that had a greater focus on connectedness within a supportive network, exhibited higher levels of blame on different relevant parties.

**Table 3:** Intercorrelations among five trusted targets at the society level (*n* = 35).

	1	2	3	4
1. Trust in God	-	-	-	-
2. Trust in a larger us	0.07	-	-	-
3. Trust in country/government	−0.12	0.12	-	-
4. Trust in science/healthcare	−0.15	0.49**	0.05	-
5. Trust in those affected	0.28	0.18	0.19	−0.00

**p < 0.01.

**Table 4:** Intercorrelations among six ways of coping at the society level (*n* = 35).

	1	2	3	4	5
1. Religious/spiritual coping	-	-	-	-	-
2. Interpersonal/social coping	0.04	-	-	-	-
3. Strength-based coping	−0.31	−0.25	-	-	-
4. Wishful thinking	0.08	0.26	−0.48**	-	-
5. Acceptance	0.14	0.18	−0.45**	0.18	-
6. Blame	−0.17	0.53**	−0.18	0.03	0.23

**p < 0.01.

#### Associations between trusted targets and coping strategies

Table 5 shows the correlations between five trusted targets and six ways of coping at the society level. To confirm the joint effects, we also performed regression analyses predicting the way of coping if there were multiple trusted targets correlated with a specific way of coping (e.g., religious/spiritual coping and wishful thinking). First, trust in science/healthcare was positively correlated with both interpersonal/social coping, *r* = 0.41, *p* = 0.016, and the focus on blaming, *r* = 0.38, *p* = 0.024, while trust in those affected was negatively correlated with the pessimistic view of acceptance, *r* = −0.39, *p* = 0.021. Trust in country/government was not associated with any coping strategies, *p* > 0.065. Second, religious/spiritual coping was positively predicted by trust in God, *b* = 0.42, *p* < 0.001, β = 0.59, but not trust in those affected, *b* = 1.80, *p* = 0.152, β = 0.20. Also, trust in a larger us was negatively correlated with strength-based coping, *r* = −0.50, *p* = 0.002, and positively correlated with wishful thinking, *r* = 0.51, *p* = 0.002. The positive association between trust in a larger us and wishful thinking was further confirmed by regression analysis in which wishful thinking was positively predicted by trust in a larger us, *b* = 6.88, *p* = 0.031, β = 0.38, but not trust in science/healthcare, *b* = 1.80, *p* = 0.104, β = 0.28.

**Table 5:** Cross-correlations between trusted targets and ways of coping at the society level (*n* = 35).

	RS	IS	SB	WT	AC	BL
Trust in God	0.64***	−0.18	−0.14	0.12	0.05	−0.29
Trust in a larger us	0.14	0.23	−0.50**	0.51**	0.30	0.21
Trust in country/government	−0.05	0.31	−0.05	0.15	0.19	0.07
Trust in science/healthcare	−0.17	0.41*	−0.16	0.47**	0.10	0.38*
Trust in those affected	0.36*	−0.08	−0.01	−0.03	−0.39*	0.03

*RS* Religious/spiritual coping, *IS* Interpersonal/social coping, *SB* strength-based coping, *WT* wishful thinking, *AC* acceptance, and *BL* blame.

* *p* < 0.05, **p < 0.01,***p < 0.001.

#### Society-level correlates of trusted targets and coping strategies

We examined the correlations of four societal factors with different trusted targets and ways of coping (Table 6). As in the above analyses, we performed regression analyses if multiple societal factors correlated with a specific trusted target and way of coping (e.g., interpersonal/social coping, acceptance, and blame). First, we found that the total number of confirmed cases was positively correlated with trust in a larger us, *r* = 0.36, *p* = 0.033, and in science/healthcare, *r* = 0.50, *p* = 0.002. The globalization index was negatively correlated with trust in God, *r* = −0.58, *p* < 0.001, while cultural tightness was positively correlated with trust in those affected, *r* = 0.66, *p* = 0.001. Individualism was not correlated with any of the trusted targets, *p* > 0.206. Second, individualism was negatively correlated with strength-based coping, *r* = −0.59, *p* < 0.001, while total number of confirmed cases was positively correlated with wishful thinking, *r* = 0.42, *p* = 0.011. Finally, although there were multiple societal factors associating with three ways of coping (viz., interpersonal/social coping, acceptance, and blame, see Table 6), regression analysis indicated that none of the societal factors were significant in predicting these coping strategies, *p* > 0.061.

**Table 6:** Cross-correlations between societal variables and trusted targets/ways of coping.

	ToC (*n* = 35)	GLO (*n* = 34)	IND (*n* = 32)	CuT (*n* = 21)
Trust in God	−0.33	−0.58***	−0.19	0.14
Trust in a larger us	0.36*	0.11	0.14	−0.33
Trust in country/government	−0.10	−0.16	−0.13	0.06
Trust in science/healthcare	0.50**	0.17	0.23	−0.15
Trust in those affected	−0.03	−0.09	−0.21	0.66**
Religious/spiritual coping	−0.17	−0.25	0.17	−0.10
Interpersonal/social coping	0.39*	0.29	0.41*	−0.39
Strength-based coping	−0.30	−0.21	−0.59***	0.31
Wishful thinking	0.42*	−0.04	0.12	−0.26
Acceptance	0.04	0.11	0.46**	−0.61**
Blame	0.36*	0.40*	0.38*	−0.22

*ToC* total number of confirmed cases, *GLO* Globalization Index, *IND* individualism, *CuT* cultural tightness.

*p < 0.05. **p < 0.01. ***p < 0.001.

## Discussion

Using a mixed-methods sequential exploratory design, the present study aimed to examine the ways that people across 35 societies managed their trust and deployed coping strategies during the early outbreak of the COVID-19 pandemic, and the roles that cultural, socioeconomic, and epidemiological factors played in the process. In this section, we will integrate the qualitative and quantitative findings, discuss implications, identify limitations, and suggest future directions.

### Integrative results

The five subthemes of trust and six subthemes of coping that emerged from the qualitative data of supportive messages were used as the core variables in the quantitative analysis. The properties of trust were further investigated with several society-level indices. The combined results show the differential effects of cultural tightness, globalization, and the severity of the pandemic on trust, which in turn influenced coping during the early stage of the COVID-19 pandemic. Our findings are discussed as follows:

#### Relationships among trust targets and relationships among coping strategies

Individuals who trust science and healthcare to lead them through the pandemic crisis are likely to attend to the public health information that the coronavirus is highly infectious and requires collective efforts at all levels to stop it from spreading. The realization that they cannot rely on themselves alone but need everyone to work together to combat this global health emergency can possibly explain the positive association between trust in science/healthcare and trust in a larger us.

When individuals hold that people should use their own inherent strengths and capacities to confront the pandemic challenges, they may be less likely to have unrealistic expectations that the pandemic will somehow subside by itself, and be less likely to support a pessimistic acceptance of the pandemic, explaining the negative association of strength-based coping with both wishful thinking and acceptance. When individuals view their interpersonal or social network as a powerful source of strength, they may see their connected others as ingroup members while blaming outgroup others for creating or worsening the pandemic situation. Viewing outgroup others as common enemies (Haller and Hoyer, 2019) serves the function of strengthening ingroup cohesion and righteousness. This may explain the positive link between interpersonal/social coping and blame.

#### Relationships between trust and coping

When individuals find themselves entrusting their fate to science and healthcare in such a huge and novel public health challenge, the feeling of being interconnected within a web of social ties in a supportive network may become more salient, explaining the positive link between trust in science/healthcare and interpersonal/social coping. Moreover, after realizing that scientists are working hard to find a cure or vaccine, and that healthcare professionals are risking their own lives to save others, individuals may feel particularly angry at those whom they think are responsible for the pandemic, explaining the positive association between trust in science/healthcare and blame. The belief that the affected have the ability to fight against COVID-19 themselves indicates a perception that the coronavirus can be defeated thus people should not surrender to it. This may explain the negative association between trust in those affected and acceptance.

The positive relationship between trust in God and religious/spiritual coping is straightforward, as the very essence of religious/spiritual coping is a trusting relationship with the higher being. A strong belief in the power and benevolence of a supernatural being can help reinforce the use of religious/spiritual coping such as positive reframing or religious rituals to transcend a stressful time into a test of faith and an opportunity for spiritual growth. This finding is in line with evidence of previous research that religious people were more likely to use religious coping (Pargament, 2001).

From an existential perspective, humans are motivated to deny their vulnerability (Becker, 1997; Sullivan et al., 2010). Trust in a larger us helps strengthen people’s sense of control in the pandemic by believing that they are not fighting it alone, but in alliance with others. However, a heavy reliance on collective efforts may undermine the importance of using one’s own strengths to cope with the pandemic challenge, explaining why trust in a larger us is negatively linked with strength-based coping. Moreover, such an inflated sense of control may also create a false sense of hope based on unrealistic optimism, explaining the positive link between trust in a larger us and wishful thinking.

#### Relationships between trust and society-level indicators

##### Epidemiology and trust

In our study, the total number of confirmed coronavirus cases was used as an indicator of pandemic severity. The higher the number of cases, the more severely one’s society was struck by the pandemic, which may also have translated into a stronger sense of urgency to control it. However, for an infectious disease of this magnitude, one cannot merely rely on one’s own effort but the collective action of others, explaining the positive link between the total number of confirmed cases and trust in a larger us. Our finding is largely in line with recent research that individuals who reported personal experience with COVID-19 (e.g., knowing someone who died from the coronavirus) also reported increased general trust, i.e., a belief that most people can be trusted (Thoresen et al., 2021). Moreover, as the novel and deadly disease requires scientific breakthrough and urgent medical care, individuals in societies that were harder hit may also more strongly trust science and healthcare to lead them through this existential threat, explaining the positive link between the total number of confirmed cases and trust in science/healthcare.

##### Globalization and trust

The negative link between globalization and trust in God might be explained by *detraditionalization*, a sociological concept referring to a consequence of globalization characterized by the awareness of alternative ways of living and the questioning of traditional beliefs (Giddens, 2003). After experiencing a rapid global exchange of information, meanings, and cultural products in the globalization process, people may be less likely to regard traditional beliefs such as religion as the only guidance for norms and values. Our finding corroborates the observation in prior research that the more globalized a society, the less religious its people (Halman and Draulans, 2006).

##### Culture and trust

To interpret the positive association between cultural tightness and trust in the affected, we may look into a key characteristic of a tight culture in expecting strict compliance to rules and regulation of its citizens (Gelfand et al., 2006; Gelfand et al., 2011), implying a fundamental belief that everyone has the inherent ability and integrity to exert self-control for the collective benefit. In the face of the collective pandemic threat, such belief may manifest itself in the trust in those affected, believing that they have the power and capacity within themselves to defeat the coronavirus and prevent it from spreading into the community. Such individual empowerment may cumulate and translate into substantial societal significance, consistent with other COVID-19 research that tighter cultures are more effective than looser cultures in combating the pandemic in terms of having fewer confirmed cases and fewer deaths (Gelfand et al., 2021).

##### Culture and coping

The negative association between individualism and strength-based coping can be understood with the concept of cultural difference in attribution (Fiske and Taylor, 1991; Ji et al., 2000), that people from individualistic cultures tend to assign the cause of social incidents to the dispositional characteristics of a person, whereas people from collectivistic cultures tend to assign the cause to the situational factors of the environment. In our study, when participants were required to show support to others affected by the pandemic, those from individualistic cultures might overestimate the dispositional causes such as low-risk awareness and insufficient pandemic preparedness of the persons, while underestimating the situational causes such as inadequate medical supplies and poor access to information in others’ social environment. Attributing the pandemic adversity to the affected may thus imply that the affected do not have the ability to combat the pandemic themselves.

##### Epidemiology and coping

For societies which were more severely hit by the novel pandemic, the changes to their people’s daily lives were possibly more drastic and the emotional toll on them might be particularly heavy. Resorting to wishful thinking might allow a temporary escape from the immense real-life adversity. This might explain why the total number of confirmed cases is positively associated with wishful thinking. Our finding is largely consistent with previous research on severe acute respiratory syndrome (SARS), another deadly disease caused by coronavirus, that perceived SARS threat was positively linked with wishful thinking coping (Lee‐Baggley et al., 2004).

### Implications

Three years into the biggest pandemic of the 21st century, with over 766 million people infected and over 6.9 million lives lost as of 24 May, 2023, the COVID-19 pandemic has shown no sign of ending (Worldometer, 2022). Though COVID-19 has become the main theme in research for the past three years, majority of the published empirical studies were quantitative in design. With its inherited limitation of set answers, quantitative research can address the “what” but not necessarily the “how” of the unique and complex contextualized experiences of people living in the pandemic. By including a qualitative phase in our study and through a supportive message writing task, we could gain an in-depth and meaningful understanding of “how” participants managed their targets of trust and ways of coping while at the same time, giving them voice and space to express themselves during the extreme isolation and uncertainty of the early outbreak. Qualitative data derived from individuals’ lived experiences were then further examined quantitatively at the broader society level to understand “what” the relationships these qualitative data were on the society-level. Using a mixed-methods design, our study captures both the “what” and “how” that people around the world under different cultural, socioeconomic, and epidemiological circumstances responded to the pandemic challenges through trust and coping. Our study sheds light on the current pandemic situation and provides lessons to learn for future global health emergencies.

Methodologically, the present research has contributed to the COVID-19 literature by using a mixed-methods design to increase both the breadth and depth of our knowledge in this unprecedented global pandemic. Our research methodology is unique in two aspects. First, due to the tedious and time-consuming nature of qualitative data collection and management, most existing qualitative research is small in size and conducted within a single culture. The magnitude of our research that encompasses 10,072 messages across 35 societies, stands out as a rare exception in qualitative literature, demonstrating the feasibility of conducting large-scale qualitative research across diverse cultural and linguistic contexts within a short timeframe to capture people’s time-sensitive responses. Second, due to the complexity of data analysis, most existing mixed-methods analyses are conducted at the individual level. The analyses of our mixed-methods research have gone beyond the individual level to include cultural, socioeconomic, and epidemiological dimensions at the society-level, illustrating the capacity of mixed-methods research in allowing more sophisticated analytic inquiry. Our work has thus provided considerable insights into the potentiality of using a mixed-methods approach in both COVID-19 and cross-cultural research.

Practically, despite methodological literature suggesting that answering open-ended questions is more time consuming and energy demanding than most closed-ended questions (Dillman, 2007), our study found that more than half of the total survey participants made use of the optional opportunity to write supportive messages to complete strangers who were affected by coronavirus, indicating that this kind of virtual connection that transcends geographical and temporal constraints might be particularly meaningful during a time of physical distancing (Elwy et al., 2021). Social organizations can build upon our approach to explore other virtual communication channels for people to maintain a sense of social connectedness during the pandemic when social distancing measures and other social restrictions are still in force. Moreover, our empirical findings in identifying the society-level predictors of trust and the relationships between trust and coping can be used by psychologists and social workers in devising psychological support interventions for those in need during and after the pandemic.

### Limitations and future directions

Although the findings of the current research can be a valuable addition to the growing COVID-19 literature, several limitations should be noted for future inquiry. First, due to design restraints, the supportive message writing task in our research was merely a one-way communication that did not allow rapport building. To further explore the dynamic and therapeutic value of this sensemaking process for both message writers and recipients, future research may consider creating a virtual forum that allows real-time and two-way interactions between participants.

Second, although various coping strategies were identified from the supportive messages in the current research, it remains unclear to what extent the strategies were used and how effective they were in dealing with the unique pandemic stress. Specifically, while there is some general consensus that certain coping strategies are more adaptive than others, it is noteworthy that the effectiveness of coping strategies is largely context-dependent. Considering the rapidly changing and ever-evolving nature of the pandemic, future research may adopt more direct and more ecologically relevant measures such as an experience sampling method (ESM), an intensive longitudinal research approach to examine the moment-by-moment temporal effects of different coping strategies on different pandemic stresses under different aversive contexts.

Third, we note that individualism-collectivism, cultural tightness-looseness, globalization, and the total number of confirmed COVID-19 cases might not be the only factors associated with trust and coping during the pandemic. As the pandemic is here to stay, future research should also explore other society-level factors such as *social axioms*, the general beliefs about how the world functions (Leung et al., 2002), which might impact who we trust and how we cope in such prolonged time of uncertainty.

Finally, our study only focused on the investigation of trust and coping at the society level by aggregating individual level data. However, it is possible for an individual’s characteristics to affect one’s choice of trusted targets and ways of coping at the individual level. Future research should examine the associations of individual characteristics with the two constructs at the individual level to establish a full picture of trust and coping in the pandemic.

## Data Availability

Data on COVID-19 severity of infection were obtained from the European Centre for Disease Prevention and Control (ECDC): https://www.ecdc.europa.eu/en/covid-19. Data on the latest version of the KOF Globalization Index were obtained from Gygli et al. (2019). Data on the cultural dimensions of individualism (vs. collectivism) and cultural tightness (vs. looseness) were obtained from Hofstede (2001) and Gelfand et al. (2011), respectively.

## Footnotes

1 We examined whether those who are willing to write a supportive message (*n* = 10,072) differed from those who are unwilling (*n* = 8099) in terms of demographic information, namely age, gender, education level and marital status. Respondents who wrote a message (*M* = 42.49, SD = 14.87) were slightly younger than those who did not write a message (*M* = 43.90, SD = 15.40), *t*(17075.09) = 6.24, *p* < 0.001. There were no gender differences across the two groups, *χ*^2^(1) = 1.13, *p* = 0.288. Respondents who wrote a message generally had a higher education level than those who did not write a message, *χ*^2^(1) = 98.56, *p* < 0.001. Specifically, among those who wrote a message, there were more people received tertiary education or above (78.2%) and fewer people received secondary education or below (21.8%) than those who did not write a message (viz., 71.8% and 28.2%). Finally, more participants who wrote a message were married (58.2%) and fewer were single, separated or widowed (41.8%) than those who did not write a message (viz., 51.4% and 48.6%).
